# Moving Beyond Mandatory Modules: Authentic Discussions About Racism and Health Equity at a Large Academic Medical Center

**DOI:** 10.1089/heq.2024.0129

**Published:** 2025-01-22

**Authors:** Karla Chamorro Garcia, Byron Gonzalez, Julia A. Healey, Leah Gordon, Maria Perla Brault, Esteban A. Barreto, Carlos G. Torres

**Affiliations:** ^1^ Cambridge Health Alliance, Cambridge, Massachusetts, USA.; ^2^ Harvard University, Cambridge, Ma ssachusetts, USA.; ^3^ CVS Health, Woonsocket, Rhode Island, USA.; ^4^ Boston College Connell School of Nursing, Chestnut Hill, Massachusetts, USA.; ^5^ Massachusetts General Hospital, Boston, Massachusetts, USA.; ^6^ Instructor of Medicine, Harvard Medical School, Boston, Massachusetts, USA.; ^7^ Instructor of Pediatrics, Harvard Medical School, Boston, Massachusetts, USA.

**Keywords:** anti-racism, diversity, equity, inclusion, storytelling

## Abstract

**Introduction:**

Our institution launched a large-scale virtual training program called “Stepping Stones” that uses allegories to provide an increased understanding of concepts, such as interpersonal, internalized, and structural racism. The goal of this project was to implement facilitated discussions with trained leaders and determine the impact of these sessions in improving the experience of the modules and boosting comfort in discussing race and racism.

**Methods:**

We developed facilitated discussions as a complimentary intervention for colleagues who participated in the virtual system-wide intervention. Our intention was to create a safe space to foster reflection and collaborative learning on how racism shows up in our work environment. We conducted 22 sessions across Massachusetts General Hospital between December 2021 and February 2023. Each session included between 5 and 30 participants who were asked to complete a survey regarding their experience.

**Results:**

We collected post-session surveys from 102 out of 350 participants. Participants found the sessions to be informative and valuable. Over 97% of respondents rated the quality of the discussions as “Excellent” or “Very Good.” Similarly, 95% of participants felt “Very” or “Somewhat” comfortable with discussing issues of race and racism in the workplace after the session.

**Discussion:**

Participants reported that the facilitated discussions were valuable, enhanced their ability to talk about racism in clinical environments, and provided an opportunity for reflection. Giving the hospital staff a common language and the ability to discuss such challenging topics may contribute to a culture of equity within our hospital.

## Introduction 

The enduring legacy of racism reverberates through marginalized communities, affecting the health and well-being of Black, Indigenous, and people of color (BIPOC) populations.^1^ As a potent social determinant of health, racism impacts health through the systematic marginalization of these communities across social, political, and economic spheres.^2^ Racism’s deep-rooted existence within the U.S. Health care system results in profound harm to both patients and health care professionals.^3^ Health care institutions and hospitals recognize the need to address systemic racism and have embraced racial equity initiatives, often through educational training targeting their employees.^1^ The current approach to addressing the need for generalizable training in anti-racism within health care relies mainly on online modules, as exemplified by the American Academy of Pediatrics in their 2021 release of an online video course on anti-racism for practicing physicians.^4^ Other notable trainings with the goal of advancing racial equity within the medical education system include implicit bias courses,^5,6^ microaggression workshops,^7,8^ and allyship sessions.^9,10^ Although laudable and important initial steps, these interventions by themselves can be insufficient. Furthermore, it is well known that continuous, ongoing training is more effective than one-time training.^11^ Additionally, these need to be led by individuals skilled in this sensitive subject area.^12^ Despite efforts to diversify academic medical centers, there continues to be a large gap in effectively including individuals from diverse backgrounds. These challenges underscore the pressing need for adept cross-cultural communication and the cultivation of an environment where diverse individuals not only find inclusion but also belonging.

An effective way to discuss complex topics, such as racism, is to invoke storytelling. Storytelling has been in use for centuries, and it can summon emotion and lead to behavior change.^13^ Storytelling has been widely studied in interventions regarding diverse health topics, including HPV vaccination and HIV treatment adherence, with positive outcomes for groups exposed to storytelling.^14,15^ Relatedly, creating a space to reflect on personal experience encourages authentic discussions and real-time input from different points of view. One such educational endeavor is “facilitated discussions,’’ which is a process that allows participants to openly share their perspective on a topic and is supported by a moderator to ensure a collaborative environment that leads to knowledge construction.^16^ Facilitators help maintain discussion structure, goals, and engagement to ensure pedagogical goals and a respectful environment that encourages reflection.^16^ Studies that have evaluated the impact of facilitated discussions have found that they improve participant engagement, enhance learning outcomes, and satisfy stakeholder expectations.^17^

As part of a multipronged antiracist campaign called “United Against Racism,” our integrated health care system launched a large-scale virtual training program called “Stepping Stones.”^18^ Four short storytelling videos were made in partnership with racism education expert Dr. Camera Jones to teach about the different levels of racism and provide a framework on how to work toward eliminating racial inequities.^19^ For example, two of the videos use the well-known works of Dr. Jones (Gardener’s Tale^19,20^ and Restaurant Saga^21^), which were enhanced for the Stepping Stones modules. Feedback from this asynchronous training suggested that staff felt as if they were completing the assigned task (or simply checking off the box) and missed an opportunity to engage as teams on these crucial conversations. Therefore, to augment and complement the online Stepping Stones modules, our team proposed and implemented optional facilitated discussions led by trained facilitators. The goal of this project was to determine the impact of these discussions in improving the experience of the modules and boosting comfort in discussing race and racism.

## Materials and Methods

### Facilitated Discussions Outline

To encourage adult learning and maximize discussion time, the sessions begin with a flipped classroom approach, where participants watch the 4 “Stepping Stones” videos introducing the discussion concepts prior to participating in the workshop. Each facilitated session, which covers two allegories discussed in the videos watched, lasts 60 min. See Table 1 for an outline of each session.

**Table 1.** Outline of Facilitated Discussion

Welcome/introductions	5 min
Ground rules	10 min
Allegory #1 discussion	15 min
Allegory #2 discussion	15 min
Debrief/Process	10 min
Close	5 min

### Setting and Participants

**FIG. 1.**
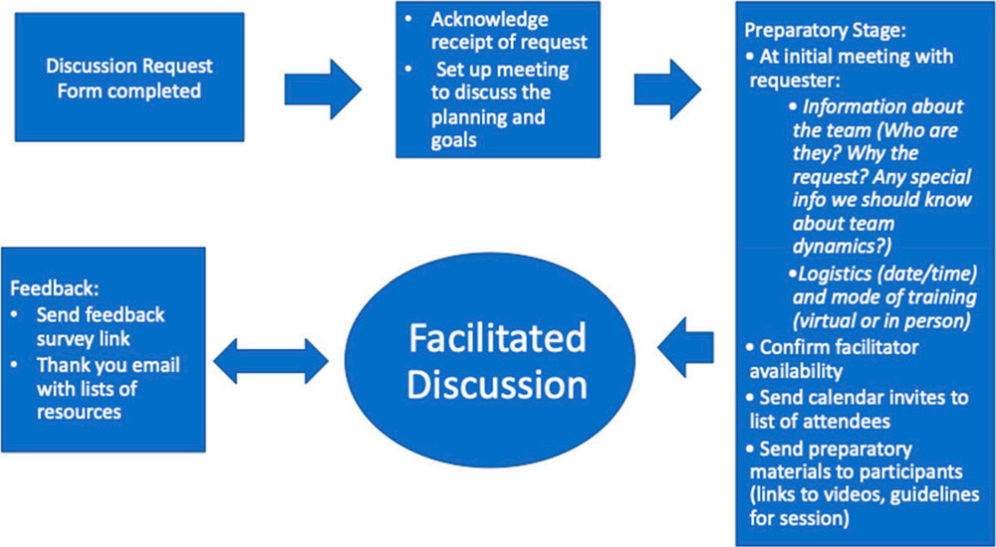
Stepping Stones Facilitated Discussion Process Map.

Watching the virtual, asynchronous videos was required for all employees at our institution and was accessed via internal hospital learning platforms. Groups and departments had the option to participate in our facilitated discussions as a complimentary program. They were invited through various mechanisms, including announcements on regular email communications. The target audience for these facilitated discussion sessions was broad and included attending physicians, nurses, social workers, and administrative personnel. We encouraged departmental teams to participate in these discussions together to foster psychological safety and allow for authentic discussions. See Figure 1 for a process map that shows the steps for setting up a facilitated discussion, which could occur in person or virtually. Our sessions took place between December 2021 and February 2023.

### Facilitators

The sessions were co-facilitated by a diverse group of health professionals from multiple role groups (physicians, nurses, social workers, and administrators). This core group was composed of eight facilitators, four who self-identified as BIPOC and four as White non-Hispanic. The facilitators were required to be comfortable leading group discussions with learners of different levels and backgrounds. They participated in a general facilitator training session, which included using a trauma-informed lens that recognizes how racism is a form of trauma that affects our bodies and minds.^22^ After this 1-h introduction, each facilitator observed two facilitated discussion sessions prior to being co-facilitators. Facilitators received a $100/h stipend for their time.

### Data Collection

We asked participants to complete a post-session survey. Main survey domains (Supplementary Appendix SA1) included level of comfort with the discussion, knowledge around race and racism, and general feedback about the session and the facilitators.

### Data Analysis

Quantitative survey data were analyzed using descriptive statistics. Qualitative data were analyzed via thematic analysis following the “five stages to qualitative research” framework.^23^

Two team members (J.H., C.T.) familiarized themselves with the data first; each read all responses to the free-form questions. They then discussed the recurring data patterns and came up with the resultant categories. These were triangulated with the quantitative survey data to identify key themes.

This project was undertaken as a quality improvement initiative at our institution and, as such, was not formally supervised by the Institutional Review Board per their policies.

## Results

We conducted 22 facilitated discussion sessions with multiple departments and programs across the Massachusetts General Hospital organization. Each session included between 5 and 30 participants. Example teams that engaged in the training included medicine (hospitalists, intensive care units, specialists such as cardiologists), emergency department, pediatrics, pharmacy, and physical and occupational therapy. A total of 350 staff members participated in our facilitated discussion sessions, and a total of 102 participants completed the post-session survey. The survey results provided data from the participants about the quality of the facilitated discussions and the facilitators and the impact of the sessions on their level of understanding and comfort discussing race and racism.

### Quality of Facilitated Discussions

Respondents remarked that the use of the powerful allegory videos made the training feel engaging and encouraged participation. One participant commented:

*“Given the topic and how challenging these discussions are, (the stories) made the topic accessible.”*

Most of the respondents rated the quality of the discussions positively (66% as “Excellent” and 31% as “Very Good)” (see Table 2). Many respondents who rated the quality of the discussion as high noted it was because the sessions were inclusive and offered a safe environment for honest conversation. One respondent remarked:

**Table 2.** Percent Respondents (*n* = 102) to Survey Question Answers

How would you rate the quality of the discussion?	*N* (%)
Excellent	67 (66%)
Very Good	32 (31%)
Good	3 (3%)
Fair	0 (0%)
Poor	0 (0%)
How would you rate the quality of the facilitators?	*N* (%)
Excellent	93 (91%)
Very Good	7 (7%)
Good	2 (2%)
Fair	0 (0%)
Poor	0 (0%)
Do you feel like you had the space and opportunity to share your experiences?	*N* (%)
Yes, definitely	86 (84%)
Yes, somewhat	16 (16%)
No	0 (0%)
Do you feel like you have an improved understanding of race, the levels of racism, and how racism may show up in your work at MGH?	*N* (%)
Yes, definitely	77 (75%)
Yes, somewhat	25 (25%)
No	0 (0%)
How comfortable do you feel discussing issues of race and racism in the workplace after participating in the facilitated dialogue?	*N* (%)
Very comfortable	23 (23%)
Somewhat comfortable	74 (72%)
Somewhat uncomfortable	5 (5%)
Very uncomfortable	0 (0%)

*“The discussion felt very open and vulnerable, which are two very important things when discussing the different levels of racism.”*

All participants (100%) reported that during the session they felt that they had the space and opportunity to share their experiences related to race and racism in the workplace. One participant stated:

*“Everyone participated and shared different perspectives.”*

Survey respondents noted that hearing these diverse perspectives and viewpoints from others added to the quality of the discussion. Participants seemed to get the most out of the discussion when attendees were willing to participate and share.

*“The depth of the sharing of perspectives on the allegories as they relate to racism and personal experiences was very well facilitated by [the facilitators] and brought out excellent discussion.”*

One area for improvement noted in the feedback included the possibility of making the discussions smaller group sizes. One participant commented:

*“The discussion was great, though I think it would have been even better if we had been in smaller groups.”*

Another potential area for improvement noted in the feedback was having more time for the discussion. A participant commented:

*“More time would be nice but I felt like we had a very fruitful discussion and didn’t feel too rushed.”*

### Level of Understanding of Race and Racism

All respondents (100%) said they had a “Somewhat” (25%) or “Definitely” (75%) improved understanding of race, the levels of racism, and how racism shows up in their work at the hospital. Participants noted that the session provided them with an increased awareness about the need to address issues of racism. Particularly, respondents who come from a position of privilege remarked on an increased understanding of racism. One participant noted how the group discussion made them recognize that:

*“I see how I come from privilege and want to continue to challenge that privilege in safe spaces in order to learn how I can impact change.”*

Respondents mentioned that the use of the allegories helped them understand the complexities of racism:

*“The allegories were helpful in explaining and illustrating complex, nuanced issues, and they were accessible ways to explore racism in our institution and ourselves.”*

### Comfort Level of Discussing Race and Racism

After participating in the sessions, 95% of participants felt “Very” or “Somewhat” comfortable with discussing issues of race and racism in the workplace.

*“This discussion gave me the confidence to speak up and name racism when I encounter it. I feel more inclined to call out disparities after attending this session.”*

Respondents also noted that the session provided them with the language and tools to discuss racism in other conversations. One participant reported:

*“I feel this was helpful to provide all of us with a common language and understanding which will make future conversations easier.”*

Generally, respondents said they felt most comfortable discussing these topics within their department or with familiar colleagues compared to engaging with staff they did not know well. One respondent noted:

*“I feel really comfortable within my department, but I’m not sure how I’d feel outside of my department.”*

The sessions helped participants understand the importance of discussing racism in order to address it. One person said that what resonated with them most from the facilitated discussion was better understanding of intervening:

*“I learned about the importance of speaking up and always questioning the status quo.”*

### Skills of Facilitators

The skills of the facilitators were an important aspect of creating high-quality discussions. A respondent noted:

*“The discussion was excellent, which was absolutely enhanced by the guidance provided by (the facilitators).”*

The quality of the facilitators was rated highly: 91% of the respondents rated the quality of the facilitators as “Excellent” and 7% as “Very Good” (Table 2). Respondents commended the facilitators’ skills in creating a safe and welcoming space by establishing ground rules for the discussion, keeping the discussion focused, motivating conversation by asking thoughtful questions, and navigating difficult comments. One respondent noted:

*“(The facilitators) truly guided our discussion, mirrored back our discussion points, and led us further into the depth of antiracism teaching.”*

Many respondents mentioned that the facilitator’s intentional use of silence encouraged people to think and share. One participant noted:

*“During our first discussion, [the facilitators] said we are not afraid of silence, and I didn’t know how impactful that could really be on a conversation. Bringing people to a space where they feel safe, supported, and challenged to think and speak their minds is wonderful.”*

Participants also remarked on how the facilitator’s knowledge and expertise on topics of race and racism added to the discussion. One participant said:

“(The facilitators’) knowledge and expertise were evident in their flow and discussion.”

## Discussion

We took a virtual, asynchronous required module and augmented it with optional facilitated discussions that allowed groups to come together and enhance their learning about racism in the health care setting. Participants found the facilitated discussions to be informative and valuable because the sessions used the power of stories as a catalyst for dialogue and provided an opportunity for learning as a community composed of interprofessional teams. Furthermore, they self-reported improvement in knowledge about racism.

Our facilitated discussions allowed for the creation of a community. Participants were able to connect with each other on a deeper level that is simply not possible with asynchronous modules. It is well documented that clinicians delivering treatments to diverse cultures often grapple with isolation and systemic barriers.^24,25^ Moreover, the lack of belonging is a known predictor of burnout, emphasizing the need for attention to cultural factors in health care settings.^26,27^ Facilitated discussions may have the potential to aid participants in finding support and motivation in their peers.^28^ An advantageous phenomenon that occurred during our sessions is that people were able to share their lived experiences, something that may be hard to do in a busy hospital setting where the focus is on caring for patients. Being in community with others allows especially marginalized learners to be seen for who they are and the daily struggles they face. Thus, our sessions allowed participants to connect with each other and come together as a community.

A critical aspect of our facilitated sessions is that we created a space in which multidisciplinary and interprofessional teams could come and openly discuss the challenges of racism in the workplace. Even though these teams work together on the same floor, cross-learning opportunities rarely happen particularly for nonclinical topics, such as racism. Most anti-racism educational interventions often target groups that are easily accessible to teachers, such as medical students and residents.^29^ However, health care is delivered by teams. Therefore, all team members must be included in these offerings. Our sessions targeted whole teams, which allowed for physicians, nurses, and administrators to come together and be aligned in how they approach situations.

The power of stories served as a key catalyst for learning and engagement with our facilitated discussions. Creating an environment that allows guided conversation and collaboration among learners has been reported as an effective strategy to increase engagement and learning.^11^ Furthermore, this approach has the potential to drive change by stimulating an emotional and empathic response that can be helpful to marginalized populations, as it serves as an opportunity to shed light on the underpinnings of their daily struggles.^30^ This in turn could be helpful in improving racial equity.

In implementing the facilitated discussions, our team learned many things. One crucial aspect was the importance of having trained facilitators. While the facilitator’s expertise and knowledge are key factors to the success of the sessions, discussions pertaining to racism must be culturally sensitive and foster openness as a tool for learners to feel comfortable to speak about their own experiences. Though our facilitators did not have to be content matter experts, they did have to be comfortable leading group discussions with a trauma-informed lens. It was also very important that they be compensated for their work and provided with administrative assistance (such as booking rooms and tech support for virtual sessions). Through our project, we were able to create a team of trained facilitators that were effective in bringing people together and stimulating generative discussions.

One challenge of bringing interprofessional teams together was finding the time for discussions to occur, especially since each role group has a different cadence to their day (front desk staff simply cannot stop checking patients into their appointments, and nurses cannot leave critically ill patients unattended). Therefore, we relied on local leadership (or the person who requested the session) to help determine the best date/time and even compensate their staff for their time if they completed these sessions outside of normal working hours. This also meant that our group of facilitators had to be flexible to accommodate these requests, including hosting these during evenings for staff who worked evenings/nights. This was only possible because we had organizational leadership support and buy-in from key stakeholders who demonstrated a commitment to this learning.

It is important to note that our project was limited by being drawn from a single institution and may not be generalizable to other settings. Additionally, our survey response rate was low; a higher response rate would ensure that it was more representative of the overall population. Selection bias may also be present whereby people with a special interest in this topic may have been more likely to participate and/or complete the post-session survey. As departments voluntarily sought facilitated discussions, we did not reach people who did not volunteer to participate. We do not know why they did not make requests to join, and they could potentially be the most uncomfortable in talking about racism. We also did not conduct pre- and post-session surveys, which may have helped in confirming change in knowledge or attitudes.

Our implementation of facilitated discussions adds value to the understanding of race and racism. For next steps, we will focus on how we can engage people and teams that have not participated in these sessions. We are also exploring how to continue working with groups who have already completed the Stepping Stones discussions and want additional learning opportunities. Given the positive results of our project, we plan to continue using storytelling as a bridge to stimulate change in attitudes and behaviors among participants to foster a culture of antiracism and equity within our hospital.

## Supplementary Material

Supplementary Appendix SA1

## Abbreviations Used

AAP: American Academy of Pediatrics
BIPOC: Black, Indigenous, and people of color
HIV: Human immunodeficiency virus
HPV: Human papillomavirus
SDoH: Social determinant of health

## References

[1] Hassen N, Lofters A, Michael S, et al. Implementing anti-racism interventions in health care settings: A scoping review. Int J Environ Res Public Health 2021;18(6):2993; doi: 10.3390/ijerph18062993 33803942 PMC8000324

[2] Czyzewski K. Colonialism as a broader social determinant of health. Int Indig Policy J 2011;2(1); doi: 10.18584/iipj.2011.2.1.5

[3] Monroe ADH, Sturgis LC, Adashi EY. Antiracism training in medicine. JAMA Health Forum 2020;1(12):e201477; doi: 10.1001/jamahealthforum.2020.1477 36218467

[4] Trent M, Johnson T, Marbin J, et al. Fighting Racism to Advance Child Health Equity. American Academy of Pediatrics online course; 2021 Apr 15.

[5] Cheng SM, McKinney CC, Hurtado-de-Mendoza A, et al. Confidence, connection & collaboration: Creating a scalable bias reduction improvement coaching train-the-trainer program to mitigate implicit bias across a medical center. Teach Learn Med 2024;36(3):381–398; doi: 10.1080/10401334.2023.2201289 37074228

[6] Gonzalez CM, Walker SA, Rodriguez N, et al. Implicit bias recognition and management in interpersonal encounters and the learning environment: A skills-based curriculum for medical students. MedEdPORTAL 2021;17:11168; doi: 10.15766/mep_2374-8265.11168 34277934 PMC8275619

[7] Calardo SJ, Kou M, Port C, et al. Realizing inclusion and systemic equity in medicine: Upstanding in the medical workplace (RISE UP)—an antibias curriculum. MedEdPORTAL 2022;18:11233; doi: 10.15766/mep_2374-8265.11233 35497676 PMC8983799

[8] Ackerman-Barger K, Jacobs NN, Orozco R, et al. Addressing microaggressions in academic health: A workshop for inclusive excellence. MedEdPORTAL 2021;17:11103; doi: 10.15766/mep_2374-8265.11103 33598543 PMC7880252

[9] Tucker Edmonds B, Neal C, Shanks A, et al. Allies welcomed to advance racial equity (AWARE) faculty seminar series: Program design and implementation. J Med Educ Curric Dev 2021;8:23821205211034940; doi: 10.1177/23821205211034940 34368456 PMC8312166

[10] Martinez S, Araj J, Reid S, et al. Allyship in residency: An introductory module on medical allyship for graduate medical trainees. MedEdPORTAL 2021;17:11200; doi: 10.15766/mep_2374-8265.11200 34988287 PMC8685188

[11] Malott KM, Schaefle S. Addressing clients’ experiences of racism: A model for clinical practice. J Counseling & Develop 2015;93(3):361–369; doi: 10.1002/jcad.12034

[12] Steed R. Attitudes and beliefs of occupational therapists participating in a cultural competency workshop. Occup Ther Int 2010;17(3):142–151; doi: 10.1002/oti.299 20641132

[13] Brooks SP, Zimmermann GL, Lang M, et al. A framework to guide storytelling as a knowledge translation intervention for health-promoting behavior change. Implement Sci Commun 2022;3(1):35; doi: 10.1186/s43058-022-00282-6 35346397 PMC8962242

[14] Lee H, Kim M, Cooley ME, et al. Using narrative intervention for HPV vaccine behavior change among Khmer mothers and daughters: A pilot RCT to examine feasibility, acceptability, and preliminary effectiveness. Appl Nurs Res 2018;40:51–60; doi: 10.1016/j.apnr.2017.12.008 29579499

[15] Lipsey AF, Waterman AD, Wood EH, et al. Evaluation of first-person storytelling on changing health-related attitudes, knowledge, behaviors, and outcomes: A scoping review. Patient Educ Couns 2020;103(10):1922–1934; doi: 10.1016/j.pec.2020.04.014 32359877

[16] Karunanayaka SP, Rajendra JCN, Ratnayake HUW, et al. Peer-facilitated discussions to enhance OER-based e-learning. AAOUJ 2016;11(1):90–104; doi: 10.1108/AAOUJ-07-2016-0022

[17] Douglas T, James A., et al; University of Tasmania, Australia. Online discussion boards: Improving practice and student engagement by harnessing facilitator perceptions. J Univ Teach Learn Pract 2020;17(3):86–100; doi: 10.53761/1.17.3.7

[18] Bryant AS, Healey JA, Wilkie S, et al. A health system framework for addressing structural racism: Mass General Brigham’s United Against Racism initiative. Health Equity 2023;7(1):533–542; doi: 10.1089/heq.2023.0077 37736521 PMC10510684

[19] Jones CP. Action and allegories. In: Racism: Science & Tools for the Public Health Professional. American Public Health Association; 2019. doi:10.2105/9780875533049ch11

[20] Jones CP. Levels of racism: A theoretic framework and a gardener’s tale. Am J Public Health 2000;90(8):1212–1215. 10936998 10.2105/ajph.90.8.1212 PMC1446334

[21] Jones C. How understanding of racism can move public health to action. Nations Health 2016;46(1):3.

[22] Kirkinis K, Pieterse AL, Martin C, et al. Racism, racial discrimination, and trauma: A systematic review of the social science literature. Ethn Health 2021;26(3):392–412; doi: 10.1080/13557858.2018.1514453 30165756

[23] Ritchie J, Spencer L. Qualitative data analysis for applied policy research. In: Analyzing Qualitative Data. Routledge; 1994.

[24] Nfonoyim B, Martin A, Ellison A, et al. Experiences of underrepresented faculty in pediatric emergency medicine. Acad Emerg Med 2021;28(9):982–992; doi: 10.1111/acem.14191 33289950

[25] Kumar S. Burnout and doctors: Prevalence, prevention, and intervention. Healthcare (Basel) 2016;4(3):37; doi: 10.3390/healthcare4030037 27417625 PMC5041038

[26] Choi SY, Kim KS. The effects of work characteristics, supervision, and cultural competence on nurses’ burnout. IJBSBT 2014;6(4):187–200; doi: 10.14257/ijbsbt.2014.6.4.18

[27] Truong M, Paradies Y, Priest N. Interventions to improve cultural competency in healthcare: A systematic review of reviews. BMC Health Serv Res 2014;14(1):99; doi: 10.1186/1472-6963-14-99 24589335 PMC3946184

[28] Carlson LM, Ridgeway JL, Asiedu GB, et al. Facilitated stories for change: Digital storytelling as a tool for engagement in facilitated discussion for reduction of diabetes-related health disparities among rural Latino patients with diabetes. J Transcult Nurs 2021;32(6):707–715; doi: 10.1177/1043659620980816 33350356

[29] Falusi O, Chun-Seeley L, de la TD, et al. Teaching the teachers: Development and evaluation of a racial health equity curriculum for faculty. MedEdPORTAL 2023;19:11305; doi: 10.15766/mep_2374-8265.11305 36999061 PMC10043344

[30] McCall B, Shallcross L, Wilson M, et al. Storytelling as a research tool used to explore insights and as an intervention in public health: A systematic narrative review. Int J Public Health 2021;66:1604262; doi: 10.3389/ijph.2021.1604262 34795554 PMC8592844

