# Supplementary material for: Moving Beyond Mandatory Modules: Authentic Discussions About Racism and Health Equity at a Large Academic Medical Center
Source: Health Equity. 2025 Jan 22;9(1):65–72. doi: 10.1089/heq.2024.0129 (PMC11773175; doi:10.1089/heq.2024.0129)
Supplement: Supplementary Appendix SA1 [file heq.2024.0129_Suppl_DataS1.docx]

**Appendix 1. Stepping Stones Facilitated Discussion Post-Session Survey**

1. Please list your department.
2. When did you participate in the Stepping Stones Facilitated Discussion?
3. Please select the Stepping Stones courses that you have already completed on HealthStream.

- Restaurant Saga
- Gardner's Tale
- Cement Dust in Our Lungs
- Tale of Two Neighborhoods
- None

1. Please select the Stepping Stones courses that you discussed through facilitated dialogue.

- Restaurant Saga
- Gardner's Tale
- Cement Dust in Our Lungs
- Tale of Two Neighborhoods

1. How would you rate the quality of the discussion?

- Excellent
- Very Good
- Good
- Fair
- Poor

1. Please explain your rating of the discussion.
2. How would you rate the quality of the facilitators?

- Excellent
- Very good
- Good
- Fair
- Poor

1. Please explain your rating of the facilitators.
2. Do you feel like you had the space and opportunity to share your experiences?

- Yes, definitely
- Yes, somewhat
- No

1. Do you feel like you have an improved understanding of race, the levels of racism, and how racism may show up in your work at MGH?

- Yes, definitely
- Yes, somewhat
- No

1. What resonated with you most from the Stepping Stones courses?
2. How comfortable do you feel discussing issues of race and racism in the workplace after participating in the facilitated dialogue?

- Very comfortable
- Somewhat comfortable
- Somewhat uncomfortable
- Very uncomfortable

1. Please tell us why you feel this way towards discussing race and racism in the workplace.
2. What additional feedback would you like to share about this experience?
